# Magnetosheath Jet Occurrence Rate in Relation to CMEs and SIRs

**DOI:** 10.1029/2021JA030124

**Published:** 2022-04-08

**Authors:** Florian Koller, Manuela Temmer, Luis Preisser, Ferdinand Plaschke, Paul Geyer, Lan K. Jian, Owen W. Roberts, Heli Hietala, Adrian T. LaMoury

**Affiliations:** ^1^ Institute of Physics University of Graz Graz Austria; ^2^ Space Research Institute Austrian Academy of Sciences Graz Austria; ^3^ Institut für Geophysik und extraterrestrische Physik TU Braunschweig Braunschweig Germany; ^4^ Hvar Observatory, Faculty of Geodesy University of Zagreb Zagreb Croatia; ^5^ Heliophysics Science Division NASA Goddard Space Flight Center Greenbelt MD USA; ^6^ The Blackett Laboratory Imperial College London London UK

**Keywords:** Magnetosheath Jets, CME, SIR, HSS

## Abstract

Magnetosheath jets constitute a significant coupling effect between the solar wind (SW) and the magnetosphere of the Earth. In order to investigate the effects and forecasting of these jets, we present the first‐ever statistical study of the jet production during large‐scale SW structures like coronal mass ejections (CMEs), stream interaction regions (SIRs) and high speed streams (HSSs). Magnetosheath data from Time History of Events and Macroscale Interactions during Substorms (THEMIS) spacecraft between January 2008 and December 2020 serve as measurement source for jet detection. Two different jet definitions were used to rule out statistical biases induced by our jet detection method. For the CME and SIR + HSS lists, we used lists provided by literature and expanded on incomplete lists using OMNI data to cover the time range of May 1996 to December 2020. We find that the number and total time of observed jets decrease when CME‐sheaths hit the Earth. The number of jets is lower throughout the passing of the CME‐magnetic ejecta (ME) and recovers quickly afterward. On the other hand, the number of jets increases during SIR and HSS phases. We discuss a few possibilities to explain these statistical results.

## Key Points

- Occurrence rate of magnetosheath jets is found to vary due to the arriving CMEs and SIRs
- Fewer jets are found when magnetic ejecta regions of CMEs hit the Earth, more jets are found when SIRs and high speed streams hit the Earth
- The jet duration does not appear to vary much during individual SW structures

## 1 Introduction

The solar wind (SW) is a continuous outflow of plasma and magnetic field from the Sun. The Earth's magnetic field is an obstacle to that SW. The SW is both supersonic and super‐Alfvénic at 1 AU. This causes the formation of a permanent standing shock wave in front of the Earth, called the bow shock where the SW is slowed down, compressed, and heated. It further evolves downstream over the magnetosheath and its inner boundary, the magnetopause, which is the dividing boundary between the Earth's magnetic field and the interplanetary magnetic field (IMF). Hence, the dynamics of the magnetosheath vary under different SW conditions (e.g., Samsonov et al., 2007; Spreiter et al., 1966).

Structures disrupting that continuous SW severely impact the bow shock and magnetopause standoff distances (Baumjohann & Treumann, 1996; Tátrallyay et al., 2012). The SW is regularly disturbed by large‐scale structures, such as stream interaction regions (SIRs) or transient events like coronal mass ejections (CMEs). SIRs are produced by the interaction between slow and high speed streams (HSSs). The fast stream often originated in open‐field coronal holes compresses the slow wind stream in front of it. This results in a compression region, where the density and total pressure increase sharply (Jian et al., 2006a). The velocity increases continuously throughout the SIR and peaks within the HSS. SIRs may periodically recur due to the Sun's rotation, which is then called a co‐rotating interaction region (CIR, Smith & Wolfe, 1976; Richardson & Cane, 2010). Other large‐scale SW structures are coronal mass ejections (CMEs), which are transient events propagating in the SW. SIRs typically present sheath‐like regions of compressed plasma and magnetic field. CMEs reveal a strong magnetic field region showing a rotating pattern in the magnetic field vector. We refer to this inner part of a CME as magnetic ejecta (ME; see e.g., Rouillard, 2011; Temmer, 2021). Because CMEs are often faster than the surrounding SW plasma, they can form a shock and drive an associated CME‐sheath region (Good et al., 2019; Kilpua et al., 2017). Typically, the energy input and the effects on Earth's magnetosphere are dominated by CMEs, especially during phases of high solar activity. On the other hand, during solar minimum and declining phase, long lived CIRs and their HSSs may continuously interact with the Earth (Tsurutani et al., 2006).

In this study, we focus on the interaction of these large‐scale SW structures with the bow shock and the magnetosheath region. Both CMEs and SIRs can compress the magnetosphere significantly due to extreme values of specific SW parameters. In particular, the SW dynamic pressure and the southward component of the IMF largely determine the standoff distance of the magnetopause (Chapman & Bartels, 1940; Fairfield, 1971; Shue et al., 1998). At the magnetopause, the dynamic pressure of the SW is equal to the magnetic pressure of the Earth's magnetic field. The place of the magnetopause is therefore a consequence of the interplay between magnetic and dynamic pressure at both sides. Large southward magnetic field values can decrease the standoff distance by reconnection processes with the Earth's day‐side magnetic field (Baumjohann & Treumann, 1996). This component is therefore considered the main driver of geoeffective interaction between the SW and the Earth's magnetic field. CMEs, SIRs, and HSSs are major sources for large southward magnetic field values (Richardson, 2018; Wu & Lepping, 2002).

While CMEs, SIRs, and HSSs arrive frequently at the magnetosheath region, they are rather sporadic events compared to so‐called magnetosheath jets. First detected in 1998 (Němeček et al., 1998), magnetosheath jets are dynamic pressure enhancements traveling downstream of the bow shock toward the Earth's magnetopause. Different names have been assigned to the same or similar phenomenon, including: transient flux enhancement (Němeček et al., 1998), supermagnetosonic jets (Hietala et al., 2012), dynamic pressure pulses (Archer et al., 2012), high‐speed jets (Plaschke et al., 2013), plasmoids (Karlsson et al., 2015), and supermagnetosonic plasma stream (Savin et al., 2014). While there are differences between each definition, they all share common properties. They either describe an enhancement in the velocity, density, or both within the Earth's magnetosheath. There is ongoing research about the origins of these jets and several generation mechanisms have been proposed, mainly involving processes at the bow shock (see Hietala et al. (2012); Karlsson et al. (2015); Preisser et al. (2020) or a review of the proposed mechanisms in Plaschke et al. (2018)). There is the consensus that the jets primarily appear downstream of the quasi‐parallel bow shock (Archer & Horbury, 2013; Plaschke et al., 2013; Raptis et al., 2020; Vuorinen et al., 2019). There is evidence that magnetosheath jets significantly influence the magnetopause and cause geomagnetic substorms in Earth's magnetosphere (Hietala et al., 2018; Norenius et al., 2021; Nykyri et al., 2019; Wang et al., 2018). Magnetosheath jets are therefore an important link between the SW and the magnetopause. Large‐scale SW structures and magnetosheath jets can be geoeffective on their own. It is therefore of great interest to learn how these effects are linked with each other.

There have been recent efforts to analyze the general favorable conditions for jet production using statistics of numerous jets (Archer & Horbury, 2013; Karlsson et al., 2015; LaMoury et al., 2021; Plaschke et al., 2013). In particular, LaMoury et al. (2021) concluded that favorable conditions for jet formation include low IMF cone angles, both slow and fast SW speeds, low magnetic field strength, high plasma‐*β*, low dynamic pressure, high Alfvén Mach number, and low density. They found that jets are more likely to survive the propagation through the magnetosheath with SW conditions showing low IMF cone angle, high SW speed, high IMF magnitude, low plasma‐*β*, and high dynamic pressure. This suggests that HSSs may have favorable SW conditions for jets, while the net effect of SIRs and CMEs cannot be deduced without dedicated research. Overall, the general relationship of jets with SW structures like SIRs, HSSs, and CMEs remain so far unexplored.

This work aims to reveal how these specific large‐scale SW structures influence the occurrence rate of magnetosheath jets. We perform a thorough statistical analysis using the overlapping times of magnetosheath observations and times of CMEs/SIRs hitting the Earth to fulfill this goal. We use magnetosheath data from Time History of Events and Macroscale Interactions during Substorms (THEMIS) spacecraft between January 2008 and December 2020. For the CME and SIR + HSS list, we use lists provided by literature and expanded on incomplete lists using OMNI data to cover the same time range. In addition, we check the robustness of our results by using two different methods for the automatized detection of magnetosheath jets.

## 2 Data and Methods

### 2.1 CME and SIR Data

In this study we use several different lists of large‐scale SW structures. We unified those lists to seamlessly cover the time range May 1996–31 December 2020.

For CMEs we use the list maintained by Richardson and Cane (Cane & Richardson, 2003; Richardson & Cane, 2010), which includes information of CMEs since 1996. It contains, among other information, start and end times for CME‐ME. It also contains the start times of corresponding CME‐shocks if one is present. We define the time between shock arrival and start of the magnetic ejecta as the CME‐sheath crossing time. The start time of the shock is defined as the time of associated geomagnetic storm sudden commencement in this list. The magnetic ejecta times are the times measured by the Active Composition Explorer (ACE, Stone et al., 1998). We briefly discuss timing issues due to measurements at L1 and the Earth in Section 4.1. The list does not include measurements of CME‐sheaths without a ME.

We use an extended collection of SIR lists to cover the time range of January 1995 ‐ December 2020. In contrast to the CME list, the definitions of start and end times of SIRs vary between different sources. We therefore made efforts to unify and standardize those lists to make our results more robust. We combine the Jian SIR list (Jian et al., 2011, time range: 1995–2009), the Grandin SIR and HSS catalog (Grandin et al., 2019, time range: 1995–2017), and the updated list by Geyer (Geyer et al., 2021, time range: 2014–2018).

The SIR and HSS list of Grandin is used as a basis for the whole list, because it provided the largest time coverage, with SIRs and HSSs from 1995 to 2017. The list provides the start time of the event, the time of maximum SW speed (within 3 days after the beginning of the event), and the end time of the event. The end time is defined by the time, where the speed drops below 450 km s^−1^ (Grandin et al., 2019). The event times of Grandin were used when an event was given in several lists.

The list by Jian provides times for each SIR, giving a start, stream interface, and end time, and the stream interface time is defined at the peak of the total perpendicular pressure (Jian et al., 2006a). For Jian's list, Wind (Harten & Clark, 1995; Wilson et al., 2021) and ACE (when Wind data is unavailable) data are used. The time of maximum SW velocity and information on the trailing HSS of each SIR is not given. We therefore manually checked each event and added the times using 1‐min resolution OMNI data (King & Papitashvili, 2005). For the time range investigated OMNI data comes from Wind and ACE at the L1 point and is propagated to the nose of the bow shock. We defined the end time of each HSS as the time when the velocity dropped below 400 km s^−1^. This value is a compromise between Grandin's list and other lists used in this paper. When several HSSs overlap and the velocity did not drop below 400 km s^−1^ in between, the time of the minimum value before the start of the next stream was used.

The list of Geyer focused on HSSs, with the start time defined as the density peak, and the end time as the time when the velocity drops below 350 km s^−1^. We manually checked that list and provided the times for the maximum velocity, the time for the velocity to drop below 400 km s^−1^, and an estimated time for the start of the associated SIR. The new start times were necessary, because the time at the density peak is usually slightly before the stream interface of the SIR. We use the start time of the SIR itself, which coincides with the increase of density and velocity.

Additionally, we manually searched for SIRs in OMNI data from 2019 to 2021, using the following definitions: the start of the SIR defined as the start of the increase of density and velocity, the maximum velocity time, and the end time where the velocity drops below 400 km s^−1^. We checked the proton temperature to gain confidence in our SIR detection, because the temperature sharply increases after the stream interface (Jian et al., 2006a). In our final SIR list, we excluded events where the velocity never reached 400 km s^−1^ and events that coincided with several or strong CMEs. These efforts ensure that we can make robust analysis of the jets happening during each type of large‐scale SW events.

For the further analysis we use the coherent lists of start and end times of the following large‐scale structures: (a) SIR + HSS, (b) CME‐sheath, (c) CM‐ME.

Table 1 shows the minimum, median, mean and maximum durations of SW events in hours. It showcases the times for SIRs + HSSs, CME‐sheaths, CME‐sheath + CME‐ME (when a CME showed both regions), and CME‐ME (all ME, regardless of the presence of a CME‐sheath). Only events that are overlapping with THEMIS magnetosheath data (see Section 2.2) are used for this statistic.

**Table 1:** Mean Durations for SW Events

	Time length (hours)			
	SIR + HSS	CME‐sheath	CME‐sheath + ME	CME‐ME (all)
Minimum	16.2	0.7	7.0	6.0
Median	87.0	10.0	33.0	20.0
Mean	100.4	10.7	36.9	23.4
Maximum	288.0	22.7	73.8	58.0

*Note.* Only events that are overlapping with THEMIS magnetosheath data are used.

### 2.2 Jet Lists

The detection of magnetosheath jets is strongly dependent on the imposed definition and thresholds. Several studies have detected jets by using dynamic pressure thresholds based on the SW (LaMoury et al., 2021; Plaschke et al., 2013; Vuorinen et al., 2019). As we analyze the occurrence of jets during SW disturbances, SW parameters (and subsequently the jet detection thresholds) can rapidly change during these times. This could cause a bias in our jet occurrence during SW events. Therefore, we compiled two lists of jets. The first jet list uses SW based thresholds, which we call the upstream jet list. The second jet list, named the local jet list, is based on local magnetosheath data to reduce the previously mentioned biases. We provide both new jet lists (upstream and local criteria) and the magnetosheath times at https://osf.io/6ywjz/ (Koller et al., 2021).

Both jet lists are created using THEMIS data (Angelopoulos, 2008). THEMIS consists of five spacecraft named A, B, C, D, and E. The orbits of the individual THEMIS spacecraft can differ and change over time, which can cause a significant difference of detected jets by different spacecraft. Therefore, we look at the data of each spacecraft individually. Because both B and C spacecraft were placed in an orbit around the Moon in 2010 as part of the Acceleration, Reconnection, Turbulence and Electrodynamics of the Moon's Interaction with the Sun (ARTEMIS) mission, we have only a small number of magnetosheath events from THEMIS B and C (Angelopoulos, 2011). We used data from the THEMIS Electrostatic Analyzer (ESA; McFadden et al., 2008) and Fluxgate Magnetometer (FGM; Auster et al., 2008). Specifically, we used the ESA ion velocity, ESA ion density, ESA temperature moments, ESA ion energy flux, and the FGM magnetic field measurements.

In order to obtain the time intervals when each THEMIS spacecraft were within the magnetosheath we used the criteria of Plaschke et al. (2013). Here we briefly describe these criteria: The spacecraft is required to be within a 30° Sun‐centered cone with tip at Earth. This ensures that the spacecraft is confined to the sub‐solar region around local noon, and therefore avoids jet criteria issues that can occur in the flanks of the magnetosheath. The distance is required to be within 7–18 R_*e*_ from the Earth's center. The measured ion density needs to be twice as dense as the solar wind. The energy flux of 1 keV ions is required to be larger than that of the 10 keV ions. This excludes times of measurements within the magnetosphere. The intervals are required to be longer than 2 min. We used the original magnetosheath interval times provided by Plaschke et al. (2013). In addition to that, we expanded the list up to 31 December 2020 by using the same criteria. Then we searched for jets in these magnetosheath intervals.

The first jet list, named the upstream jet list, uses the criteria given by Plaschke et al. (2013). The main threshold is given by $p_{\text{dyn,}x}>\frac{1}{2}p_{\text{dyn,}\text{x,}\text{sw}}$, using upstream SW data from 1‐min resolution OMNI data at the same time as a base for setting the threshold. *p*_dyn, x_ denotes the dynamic pressure in GSE‐X direction, and *p*_dyn,x,sw_ the dynamic pressure of the SW in GSE‐X direction. The time range for the jet was then defined as the range when the dynamic pressure exceeds 1/4 of the SW dynamic pressure. We used the original list of jets from 2008 to 2012 for THEMIS A‐E by Plaschke et al. (2013) and the expanded list of jets using THEMIS A, D, and E from 2012 to 2018 (LaMoury et al., 2021; Plaschke et al., 2013). Both original lists are available online (Plaschke, Hietala, & Angelopoulos, 2020; Plaschke, Hietala, & LaMoury, 2020). We reforged the jet list to include the time range of 1 January 2018–2031 December 2020. It is important to note that THEMIS data are sometimes reprocessed. Therefore there might be differences in the jets and magnetosheath times between the current list and the original datasets.

Our second jet list, which we name the local jet list, uses the following criteria: $p_{\text{dyn,}x}>3\times {\langle p_{\text{dyn,}x}\rangle }_{20\text{min}}$. Here, ${\langle p_{\text{dyn,}x}\rangle }_{20\text{min}}$ denotes the 20 min running average of the magnetosheath dynamic pressure in GSE‐X direction. All magnetosheath times shorter than 20 min (e.g., close to the boundary) are not considered. This definition is a modification of the jet definition used by Archer and Horbury (2013), but we use the component of the dynamic pressure in the GSE‐X direction similar to the upstream jet list definition. Archer and Horbury (2013) used a factor of 2 as a threshold for the dynamic pressure. Because we only use the GSE‐X velocity component (which is the most significant component in the magnetosheath), we settled on using the next higher integer as a threshold. The time range for the jet was then defined as the range when the dynamic pressure increases above $2\times {\langle p_{\text{dyn,}x}\rangle }_{20\text{min}}$. This resulted in a jet list from start of January 2008 to December 2020 for THEMIS A, D, and E and January 2008 to December 2009 for THEMIS B and C.

**Figure 1:**
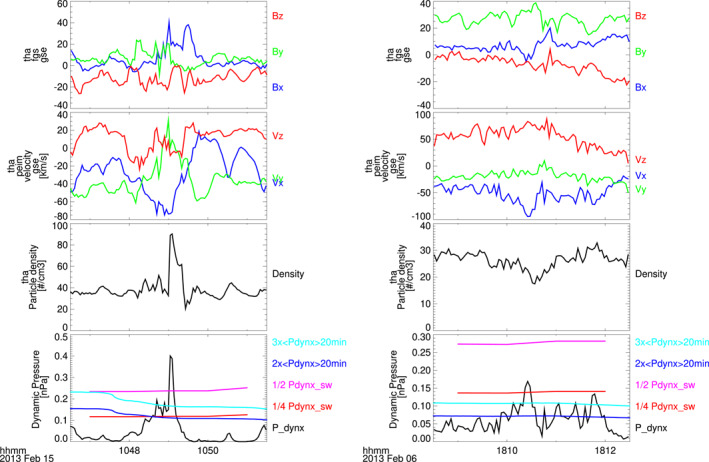
Two examples of jet detection by THEMIS A with threshold comparison. From top to bottom: magnetic field components, ion velocity components, particle density, and dynamic pressure. The dynamic pressure thresholds for both jet definitions are displayed in each bottom panel. The local definition thresholds (turquoise and blue) are defined as 3 and 2 times the 20‐min‐averaged magnetosheath dynamic pressure in GSE‐X direction. The upstream definition thresholds (pink and red) are defined as 1/2 and 1/4 times the SW dynamic pressure in GSE‐X direction. In the case shown on the left side, the lower dynamic pressure thresholds of both conditions (red and blue), which mark the beginning and end of the jet, are almost identical, while the upper threshold, marking the dynamic pressure that must be exceeded for the detection, is higher for the upstream condition (pink). In the case shown on the right side, the upstream jet conditions did not detect any jets, because the detection threshold (pink) is too high, while the local jet criteria (cyan) detected two jets.

The original upstream jet list used the dynamic pressure in *x* direction only to mainly find jets that can reach the magnetopause. We followed up on this goal in our definition for the local jet list. As a positive side effect, both lists became comparable. This validates that we are indeed looking at the same jet effects. To ensure this, the local jet list includes the same side criteria as the upstream jet list (Plaschke et al., 2013): the ion GSE‐X velocity of the jet has to be negative, and the magnetosheath GSE‐X velocity within 1 min before and after the jet interval has to go above half of the measured GSE‐X velocity during the jet's dynamic pressure peak. Calibration features and orbit differences might impact the total number of jets detected for individual spacecraft. We manually checked to make sure that the detected jets are indeed distinct pressure enhancements over the background value for each spacecraft. Figure 1 shows the differences between both detection criteria for two examples. Following this procedure we obtain a different number of jets that is summarized and compared in Table 2. For each jet list we give the number of jets detected by each spacecraft, the total jet time in days as well as the mean and median jet time in seconds. The last row shows, how many jets of the list are (at least partially) overlapping with jets from the other list. The difference in the number of overlapping jets stems from the fact that several jets in a list may overlap with only one jet from the other list.

**Table 2:** Statistical Overview of the Two Main Jet Lists Used Within This Work

	Upstream jet list	Local jet list
Total jets	16,494	18,808
THEMIS A	4147	5405
THEMIS B	147	118
THEMIS C	586	506
THEMIS D	3801	5001
THEMIS E	7813	7778
Total jet time (days)	8.7	6.2
Mean jet time (sec)	45.6	28.5
Median jet time (sec)	29.0	19.0
Number of overlapping jets	8935	9351

Orbits of the different spacecraft may be similar, which could result in single jets detected at more than one spacecraft. We give spacecraft separation estimates for THEMIS A, D, and E to address the issue of double‐counting of jet events. We derive that for 39.77% of the available observation time, only one of the three spacecraft was within the previously defined magnetosheath range. We determine the spacecraft separation for the residual time, which means for all instances when at least two spacecraft were within the defined magnetosheath range at the same time. As jets dominantly move along the GSE‐X direction, we determine the separation in the GSE y‐ plane. The average Y‐Z separation for all instances over the whole time range was 1.33 R_*e*_ with a standard deviation of 1.25 R_*e*_. We find that the orbits of the THEMIS spacecraft changed significantly during the analyzed time range. The orbits deviated from each other in the time range of 2016–2019. We determine an average Y‐Z separation of 2.54 R_*e*_ with a standard deviation of 1.59 R_*e*_ for this time range. Overall, all three spacecraft together showed the closest separation in 2010 with an average distance of 0.40 R_*e*_ and a standard deviation of 0.15 R_*e*_. Considering this, the average separation of THEMIS spacecraft exceeded the expected median perpendicular scale size of jets of 0.12 R_*e*_ (see Plaschke et al., 2020) during the analyzed time range. We conclude that most of small and medium sized jets got detected by a single spacecraft. Large jets might get detected by two or more spacecraft during times of little separation.

### 2.3 Analysis Methods

In order to study how the jet occurrence behaves during large‐scale SW events, we follow a three‐step procedure as described in the following.

Step 1: Quantifying the amount of available data. We checked the total time of magnetosheath observations as well as the number of jets that overlap with times of large‐scale SW structures (SIR + HSS, CME‐sheath, CME‐ME). Little overlap of magnetosheath data with SW disturbances lead to high uncertainties in the subsequent analysis. To determine whether the duration or number of jets is changed during disturbances, we quantify the jet mean and median time length for each type of event. We visualize the distribution of jet durations for each type of disturbances as well as quiet SW times (all times where neither SIR nor CME interacts with Earth) by using boxplot statistics.

Step 2: First order estimate of jet occurrence rate during CME and SIR times. We define a “jet percentage” during a specific time range, given by the total duration of jet time divided by the total duration of magnetosheath measurement within that given time range. This is calculated for all SIRs + HSSs, for all CME‐sheaths, and for all CME‐MEs. We also calculate the jet percentage during quiet SW time, and over the entire available time range (including both quiet SW times and times of SW structures), which we call the “overall jet percentage”. The values are given individually for each spacecraft, to cross‐check for instrumental and orbital effects. We also calculate the mean number of measured jets per hour to check, how the value for each type of event is changing compared to the jet percentage. The jet percentage is codependent on the size and speed of jets, while jet occurrence does not take that into account. We mainly focused on the jet percentage to make conclusions based on the total jet observation time. In addition to that, the jet percentage is not drastically influenced by short jets that barely meet our defined threshold. This makes the results more robust against uncertainties in the jet criteria definition.

Step 3: In detailed analysis of jet occurrence during CME and SIR times. We used a superposed epoch analysis (SEA) to determine at which time in the CME or SIR profile the jet occurrence rate changes. For SIRs + HSSs, we set the zero epoch, that is, 0 hours, at the start of the SIR (defined as the onset of the velocity and density increase) and the end time to the mean SIRs + HSSs duration in hours (see Table 1). For CME‐sheath and CME‐ME, we use a 3‐point SEA to analyze both parts of the CME separately. The length of each individual event varies largely, therefore we have normalized each CME‐sheath and CME‐ME to their respective mean duration (see Table 1). We set the zero epoch for the CME‐sheath to be the CME‐shock arrival time and its end to the mean time length for CME‐sheath (11.7 hr, see Table 1). The arrival of the CME‐ME marks the zero epoch time for the CME‐ME part. It ends after the mean time length of all associated CME‐MEs. Both SEA are then joined together where the CME‐sheath time ends and the CME‐ME begins to form the 3‐point SEA. The mid‐point time of magnetosheath intervals and jet intervals are converted to the new SEA timeline. The individual jet duration as well as most sheath measurements are short compared to CME and SIR timescales. Therefore, we bin the time axis in 1 hr duration bins and sum up the duration of each jet and sheath in the associated bin. Each interval is summed up in the bin in which the interval mid‐point falls in the new SEA timeline. The original sheath and jet interval durations are used for the sum in each bin. Otherwise, intervals measured in short SW structures would be stretched and over‐represented. Intervals during shorter structures would have been compressed and thus under‐represented for each bin, the jet percentages are calculated. The jets are sporadic events, therefore, a running average of the final percentage per time is necessary. We apply a running average using a sliding window with a length of 50 hr for the SIRs + HSSs and 10 hr for the CME‐sheath + ME plots. We applied the SEA for SIR + HSS and for CME‐sheath + ME. CME‐MEs without a sheath are not analyzed using SEA because of the small number of available events. Only CMEs that show both a sheath and a ME were considered to find conclusions for both individual parts of the structure.

The final result yields a jet percentage time evolution for the mean CME‐sheath + ME and SIR + HSS structures. We used a bootstrapping approach to check the robustness of the result and to give very conservative error estimates. We redo the analysis and randomly select (and replace) a sample covering only 50% of all sheath observations for each spacecraft. We repeated this 100 times for each event type, resulting in 100 different profiles of jet percentage evolution and their related mean jet percentages. The standard deviation of the derived jet percentages are given as uncertainties. This method puts the results from the second step into perspective and enables us to make general conclusions on the temporal evolution of jets during SW structures. We compare the jet percentage evolution with the quiet jet percentage that we defined in method. We used the bootstrapping method to get an error estimate for the mean quiet value as well.

We address the results of each spacecraft individually. By not mixing the jet results, we can make clear statements and conclusions about the relative change in detected jets for different solar wind time periods for each spacecraft, independent of possible calibration or orbital differences. With that we also avoid the possible issue of double‐counting jets that might have been detected by several spacecraft due to times of similar orbits.

**Figure 2:**
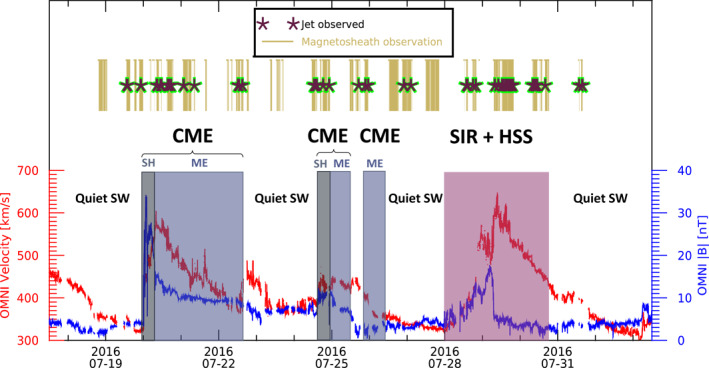
Timeline plot of July 2016 showing an example of observed jets by THEMIS A, D, and E (indicated as star symbols) during CME sheath (SH), CME magnetic ejecta (ME), SIR and quiet SW times. Time ranges of available magnetosheath observations by any spacecraft are plotted in gold. The bottom panel shows OMNI total velocity and total magnetic field during the time range.

Figure 2 shows the visualization of a time range to give an example of the available data. We have magnetosheath observations by THEMIS overlapping with both CME and SIR structures hitting the Earth in the given time range. Observed jets, which are very short in time compared to the displayed time range, are displayed as stars in this figure. The CME structures are divided into the CME‐sheath and the CME‐ME. To show the SW conditions, the OMNI data for the total velocity and the total magnetic field is plotted. The CMEs show a distinct strong magnetic field, while the SIR and HSS show the typical profile of high SW velocity over several days.

## 3 Results

### 3.1 Step 1 Results

Table 3 shows the total time (given in days) of available magnetosheath data during each type of SW events. The number of individual events is also given. The results are highly influenced by the orbits of each spacecraft. THEMIS B and C show only little magnetosheath dwell time overall compared to the other spacecraft. There is almost no magnetosheath observation during CMEs for both spacecraft. Therefore the focus in the further statistics are put on the spacecraft A, D, and E.

**Table 3:** Total Time (in Days) of Magnetosheath Observation by Each Spacecraft During Each Type of Events

	Observation time in Magnetosheath (# of individual events)			
	Total time	SIR + HSS	CME‐Sheath	CME‐ME
	(days)	(days)	(days)	(days)
THEMIS A	156.3	52.6 (85)	3.4 (28)	9.0 (49)
THEMIS B	3.4	1.0 (12)	0.1 (1)	0.1 (1)
THEMIS C	11.1	3.8 (18)	0.0 (1)	0.0 (0)
THEMIS D	127.8	42.4 (83)	3.8 (29)	8.4 (45)
THEMIS E	157.9	54.7 (87)	3.3 (25)	9.9 (47)
Total	456.6	154.5 (105)	10.6 (39)	27.4 (55)
Percentage of total time	100%	33.8%	2.3%	6.0%

*Note.* The number of individual SW events that overlap with magnetosheath measurements are given in parentheses.

Table 4 and Table 5 show the number of detected jets during each type of events for the upstream jet and the local jet list, respectively. THEMIS B and C show fewer detected jets compared to the other spacecraft, which is a result of the little magnetosheath dwell time. With several thousand jets, we observed the most jets during SIR and HSS structures. Comparing with the total number of detected jets, we see that roughly 40% of all jets are observed during SIRs and HSS times. This is valid for all spacecraft surveyed. The number drops by an order of magnitude when looking at the CME‐sheath revealing roughly 100 observed jets for each spacecraft. In comparison, the number of jets increases slightly for the CME‐ME times, with a maximum of 316 jets for THEMIS E. We see that in both jet lists, THEMIS E shows the most jets of all five spacecraft.

**Table 4:** Number of Detected Jets During Large‐Scale SW Events for the Upstream Jet List

Upstream jet definition	Total	Jets during SIRs + HSS	Jets during CME ‐ sheath	Jets during CME ‐ ME
THEMIS A	4,147	1,783	70	86
THEMIS B	147	53	2	1
THEMIS C	586	216	0	0
THEMIS D	3,801	1,563	106	107
THEMIS E	7,813	3,705	114	199

**Table 5:** Number of Detected Jets During Large‐Scale SW Events for the Local Jet List

Local jet definition	Total	Jets during SIRs	Jets during CME ‐ sheath	Jets during CME ‐ ME
THEMIS A	5,405	2,184	96	236
THEMIS B	118	59	1	0
THEMIS C	506	200	0	0
THEMIS D	5,001	2,241	109	188
THEMIS E	7,778	3,562	118	316

**Figure 3:**
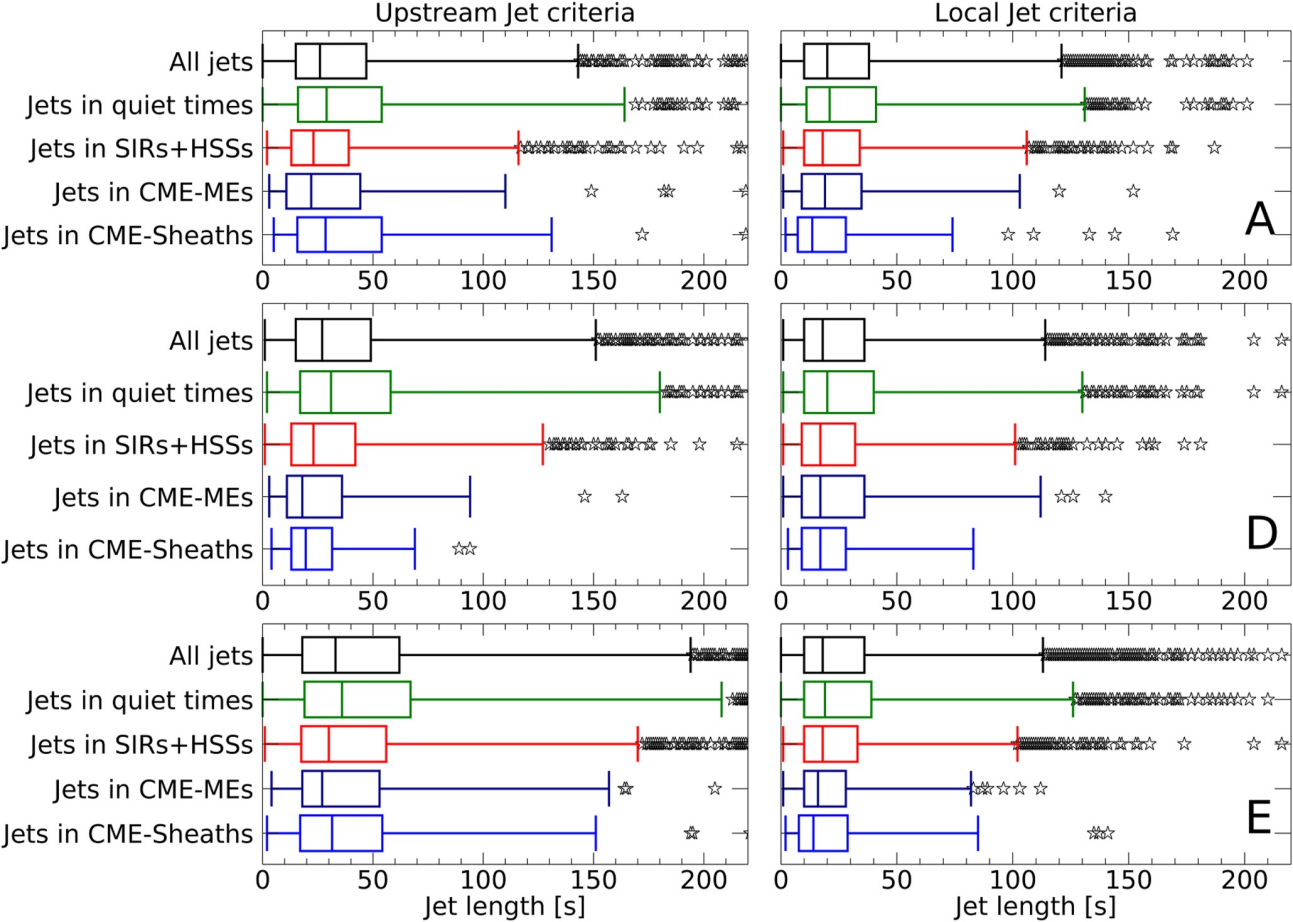
Statistical boxplot for the upstream and local jet lists, giving the jet duration for all jets and for jets that occurred during SW quiet times, SIRs + HSSs, CME‐sheaths, and CME‐MEs. The rows, from top to bottom, correspond to data from THEMIS A, D, and E. Each box shows the interquartile range. The middle line in the box shows the median length of jets in each case. The outliers, which are represented by black stars, are defined as all values beyond three times the length of the interquartile range.

Next, we calculate the mean and median duration of jets during SIRs + HSSs and CMEs. This helps to determine, whether the production or duration of the jets is more affected by each type of event. Figure 3 shows the distribution of the jet time length for each event using box plots for the upstream jet and local jet definition. The box shows the interquartile range, which is the range between the first and the third quartile. Therefore, 50% of the jet lengths are within the box. The middle line in the box shows the median length of jets in each case. The whiskers show the upper and lower limit of the distribution. Outliers are defined as all values beyond three times the length of the interquartile range. They are displayed as black stars in the plots. The median values and interquartile ranges for jets during SIRs + HSSs, CME‐sheaths and CME‐MEs are fairly comparable for each spacecraft and jet definition. The jet lengths between spacecraft are more comparable using the local jet criteria. The duration of jets during CME‐sheaths tend to be shorter compared to the other structures in the local jet criteria. On the other hand, the duration of jets during quiet SW times seem to slightly exceed the jets during structured SW. In general, the interquartile ranges overlap in every category. We see that the range of outliers is drastically greater for the upstream jet definition and go far beyond the displayed range here. For each boxplot, the number of outliers range between 2% and 4% of the total number of detected jets. The number of outliers appear to be proportional to the number of detections and not dependent on the type of event. Overall, we see that the duration of jets are not drastically influenced by different SW structures. Therefore, the results calculated in step 2 and step 3 are primarily influenced by the number of jets produced during SW structures.

### 3.2 Step 2 Results

The resulting jet percentage and the mean number of jets per hour during specific time ranges (all times, quiet SW, SIR + HSS, CME‐sheath, CME‐ME) is shown in Table 6 for the upstream jet definition and in Table 7 for the local jet definition. As previously mentioned, we differentiate between results for THEMIS A, D, and E. The difference in jet percentage between the spacecraft is smaller for the local jet definition. Overall, the range of values for the local jet list is significantly smaller compared to the upstream jet list. The percentages of jets during quiet SW conditions are fairly comparable with the overall mean values. The jet percentage for THEMIS E exceeds both other spacecraft in every category for both jet definitions. We suspect that a calibration feature may cause this difference in the data.

**Table 6:** Mean Jet Percentages and Jets Per Hour During Each Event Type for the Upstream Jet List

	Jet percentages—upstream jet criteria				
	Overall	Quiet SW	SIR + HSS	CME‐sheath	CME‐ME
THEMIS A	1.19%	1.22%	1.26%	1.24%	0.56%
THEMIS D	1.39%	1.54%	1.37%	0.82%	0.44%
THEMIS E	2.96%	2.75%	3.69%	1.81%	1.35%
	Jets per hour—upstream jet criteria				
THEMIS A	1.1	1.0	1.4	0.8	0.4
THEMIS D	1.2	1.2	1.5	1.2	0.5
THEMIS E	2.1	1.8	2.8	1.4	0.8

**Table 7:** Mean Jet Percentages and Jets Per Hour During Each Event Type for the Local Jet List

	Jet percentages—local jet criteria				
	Overall	Quiet SW	SIR + HSS	CME‐sheath	CME‐ME
THEMIS A	1.18%	1.17%	1.28%	0.79%	0.80%
THEMIS D	1.27%	1.23%	1.51%	0.72%	0.72%
THEMIS E	1.60%	1.51%	1.93%	0.98%	0.85%
	Jets per hour—local jet criteria				
THEMIS A	1.4	1.3	1.7	1.2	1.1
THEMIS D	1.6	1.4	2.2	1.2	0.9
THEMIS E	2.1	1.8	2.7	1.5	1.3

We find that, in general, the percentage as well as the number of jets per hour is increased while a SIR + HSS is passing the Earth. Exceptions are found in the upstream list for THEMIS A and D, where the SIR + HSS percentage is close to the overall value. However, the number of jets per hour is still increased in both cases. The increase of jets per hour for SIR + HSS times is roughly between 20% and 50%. For CME‐sheath times, we see a general trend of a jet percentage and jets per hour drop. Only THEMIS A in the upstream jet list shows no drop in the CME‐sheath compared to the mean value. However, the number of jets per hour still decreases. The drop in jets per hour is roughly between 0% and 30%. For the CME‐ME times, we see a clear drop of jet percentage and jets per hour for every spacecraft for both jet definitions. The drop in jets per hour is roughly between 20% and 60%.

The following trend is visible for all spacecraft in both definitions: jet percentage during SIR + HSS ≥ jet percentage during CME‐sheath ≥ jet percentage during CME‐ME. The same findings hold for the calculated jets per hour.

### 3.3 Step 3 Results

**Figure 4:**
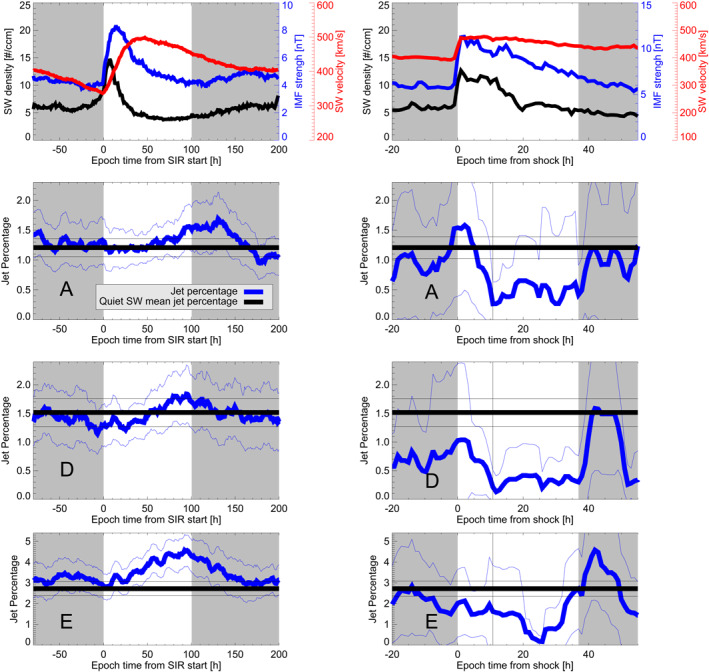
Mean SW parameters (first row) and jet percentages for THEMIS A, D, and E (row 2–4) using the upstream jet definition. The left column shows the values for the SIR + HSS times, the right column shows the values for the CME‐sheath and CME‐ME times. The mean SW velocity (black), IMF strength (blue), and SW density (red) is plotted. The jet percentages are plotted using a bold blue line. The faint blue lines are the error estimations. The bold black line shows the quiet mean value (Table 6) and the faint black lines show the error estimations.

**Figure 5:**
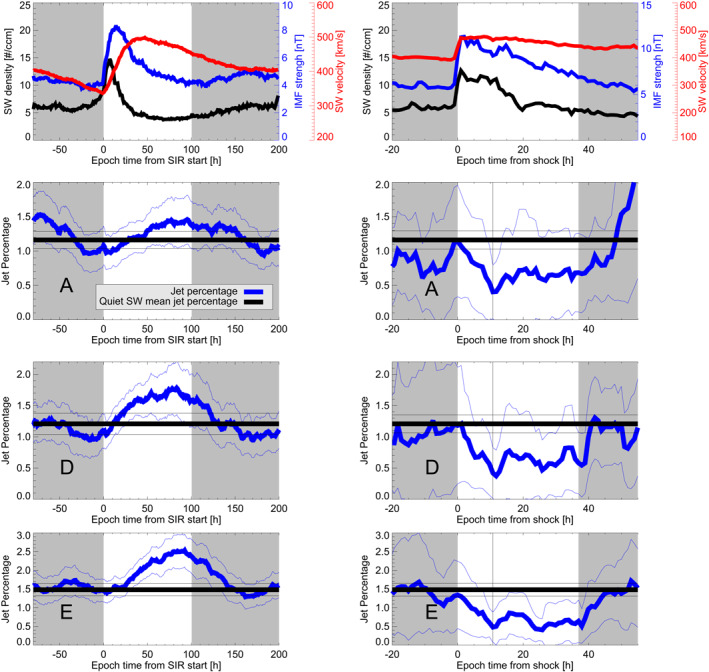
Same as Figure 4 but for the local jet definition.

The evolution of the jet percentage over the mean SIR + HSS and CME‐sheath + ME times is shown in Figure 4 and Figure 5 for the upstream and local jet list, respectively. The mean SW parameters during SIR + HSS and CME‐sheath + ME are plotted in the first row. The second row of each figure corresponds to THEMIS A data, the second row to THEMIS D, and the third row to THEMIS E. The mean jet percentage value of the quiet SW is plotted for comparison in black. The results for SIRs + HSSs are shown in the left column. The jet percentage at the start of the SIR roughly coincides with the mean quiet value. The jet percentage shows an increase after the SIR start. This finding is valid for each spacecraft surveyed for both jet definitions. The peak of the jet percentage is visible at roughly 75–90 hr after the zero epoch for most spacecraft. Only THEMIS A in the upstream jet criteria shows the peak after the end of the HSS. The decrease in percentage seems to continue after the defined ending of the HSS. The associated uncertainties are lower compared to the CME results, because the number of available SIRs that overlap with magnetosheath data is larger.

The results for CME‐sheaths + MEs are shown in the right column of Figures 4 and 5. Each jet percentage datapoint in both figures is equivalent to a 1‐hr bin. On average, each bin has roughly 12 hr of magnetosheath data during SIRs and 8.5 hr of data during CME‐sheath and CME‐ME. The jet percentage during the CME‐sheath time is monotonically decreasing for each spacecraft surveyed for both jet definitions. The jet percentage during the CME‐ME is lower than the mean value for each spacecraft surveyed for both jet definitions. The jet percentages recover sharply after the end of the CME‐ME. The estimated uncertainties are higher compared to the SIR SEA. This is the result of the low number of CME‐sheaths + MEs that overlap with Earth's magnetosheath measurements, as was previously mentioned. In addition to that, the restriction to analyze each spacecraft individually enlarges the uncertainty for each single analysis. Still, every spacecraft shows the same general trend within the SW structures in each analysis. This improves the confidence in our results.

When we compare the jet percentages of SIR + HSS, CME‐sheath and CME‐ME profiles with each other, we see the same picture over all spacecraft and jet definition: Jet percentages start to rise strongly during the SIR passage reaching a peak after the HSS reached its maximum speed. The jet percentage is decreasing sharply during the passage of the CME‐sheath with low values close to the transition from sheath to CME‐ME structure. During the entire CME‐ME time, the percentages stay at a low level and recover as the CME‐ME structure ends.

## 4 Discussion

### 4.1 Diminished Jet Numbers During CME Passing

Previous studies found a clear correlation of jet production downstream of Earth's bow shock with a steady IMF that is quasi‐parallel to the bow shock normal (Archer & Horbury, 2013; Plaschke et al., 2013; Vuorinen et al., 2019). The IMF usually becomes highly variable during CME‐sheaths (e.g., Jian et al., 2006b), which could disrupt a stable foreshock. This in turn results in fewer jets that get produced. On the other hand, the highly dynamic plasma in the CME‐sheath may cause a new rippling in the bow shock. In our study we derive, regardless of spacecraft, that the jet percentage is clearly dropping during the passing of the CME‐sheath (see Figures 4 and 5). Further analysis on a case‐to‐case basis of these regions will enable us to better understand the physical processes behind.

The IMF angle drastically changes within the CME‐ME, and hence, the position of the quasi‐parallel shock front (and the foreshock). However, the timescale of the changing IMF angle is much longer (several hours) compared to the timescale of jet generation (several minutes). The IMF in the CME‐ME is steady for timescales of roughly 10 min, which is expected to be a favorable condition for jet production. This might indicate that the presence of a strong IMF itself is a key factor that inhibits jet generation. We find in our study that the number of jets is very much lowered during the CME‐ME but still covers a significant number of jets. We may speculate that these jets are different compared to the jets observed during quiet SW times as the bow shock region where jets get produced might change during the CME passage. Raptis et al. (2020) performed statistical analysis of jets and differences in their parameters downstream of the quasi‐parallel and quasi‐perpendicular shocks. They concluded that jets downstream of the quasi‐parallel shock front occur more frequently and possess higher dynamic pressure and duration compared to jets found downstream of the quasi‐perpendicular shock. They also noted the existence of “encapsulated jets”, which show properties similar to quasi‐parallel jets but are found behind the quasi‐perpendicular shock front. Raptis et al. (2020) suggested that these jets may originate from the flanks of the bow shock during large IMF cone angles. Further investigation might reveal whether we see the same effect in the properties of jets that are observed during the CME‐ME.

From our detailed analysis using SEA, we find that the number of jets seem to recover as the CME‐ME ends. The wakes of CMEs might possess radial IMF for an extended period of time (Neugebauer et al., 1997), which would benefit the production of jets. However, at this point we did not exclude multiple CME events (this would have lowered our statistics). We infer that the SW conditions in the trailing region of the CME might play an important role in the jet production rate. As sequences of CMEs would change these conditions, they should be taken into account. Hence, the shown results are inconclusive whether the sharp increase of jets after the CME is due to favorable SW conditions or due to the recovering of the mean jet production rate.

A constant extremely high dynamic pressure level within the individual parts of the CME (especially sheaths) may cause non‐detection, because the jet detection threshold could be increased beyond the usual dynamic pressure value of jets. On the other hand, we find in our study an increase of jet percentage during SIRs, which is related to a moderately higher dynamical pressure too (Jian et al., 2006a). The effect of CMEs compressing the bow shock and the magnetopause (Sibeck & Gosling, 1996) has not yet been considered in the statistics. This could cause the spacecraft to temporarily change the position within the magnetosheath regarding the distance to the bow shock. Because jets are more frequently observed in the close proximity to the bow shock, this plays a role in studying jet statistics (LaMoury et al., 2021; Plaschke et al., 2013; Vuorinen et al., 2019). There are three possible outcomes of this compression regarding the relative position of the spacecraft: First, the spacecraft is positioned within the magnetosheath and the distance to the bow shock shrinks during compression. This would cause an increase in detected jets. Second, the spacecraft is close to the bow shock and crosses the shock during the compression, causing the spacecraft to be in the SW. This would first lead to an increased number of jets at the beginning, and a decrease in sheath data during the compression. Third, the spacecraft is within the magnetosphere close to the magnetopause, and the compression causes the spacecraft to cross the magnetopause, causing the spacecraft to be within the magnetosheath. This would lead to no sheath data at the beginning, and low jet numbers after during the compression. However, the mean time that THEMIS spacecraft spend in the magnetosheath during each revolution around the Earth is several hours shorter compared to the duration of most SW structures. This suggests that the positioning in the magnetosheath might be more affected by the orbit of the spacecraft even during a simultaneous compression of the magnetosphere. A case‐by‐case future study could help to study effects in detail.

The list by Cane and Richardson (2003); Richardson and Cane (2010) uses times for the ejecta part measured by ACE at L1 rather than the arrival time at the Earth. We find that this issue has little influence on our statistics and no change on our general conclusions. The time shift is expected to be roughly in the range of 1 hr, which is rather small compared to the mean length of the ME (between 20 and 30 hr, Table 1). The influence on the SEA results are also negligible because the running average window is significantly larger than the time shift.

### 4.2 Increased Jet Numbers During SIR + HSS Passing

A fast SW appears to be somewhat correlated to a higher numbers of jets according to LaMoury et al. (2021). Specifically, LaMoury et al. (2021) found that both slow and fast SW are beneficial for jet generation at the bow shock, and jets are more likely to reach the magnetopause during high SW velocities. Overall, fast SW appears to be a favorable factor for the number of jets found within the magnetosheath. Our results of enhanced jet percentages during SIR + HSS passing agree with these results. We clearly observe that the jet percentage monotonically increases after the zero epoch (defined as onset of the SIR velocity and density increase), independent of jet definition and spacecraft surveyed. The maximum of the jet percentage is reached after the maximum speed during the HSS is reached, hence, close to the defined end of the HSS (see Figures 4 and 5). This corresponds to mean SW conditions with low density, low IMF strength, and high (although decreasing) SW velocity. The percentage reaches mean values roughly 50–75 hr after the defined end of the HSS. At this time, the SW conditions are also supposed to be back to quiet mean conditions.

Similar to the CME times, the effect of SIRs compressing the bow shock and the magnetopause has not yet been considered in the statistics. In principle, the same impacts that we previously discussed in Section 4.1 apply. Both SIRs + HSSs and CMEs have compressing effects on the bow shock and magnetopause. In particular, SIRs and CME‐sheaths often show very similar SW parameters that can affect the standoff distances (rapidly changing IMF strength and direction, velocity and density increase), but they show the exact opposite effects in the jet percentage. This rules out the possibility that the results are mainly caused by differences in the compression of the bow shock and magnetopause. There is a difference in the time profiles of increased dynamic pressure for both types of events, but both timescales are significantly longer than the timescales expected for jet generation.

### 4.3 Different Jet Definitions

The number and time length of detected jets vary significantly depending on the definition. The jet threshold based on upstream conditions can be a source for errors when sudden events are impacting the Earth. This would suddenly change the jet threshold and therefore bias our results during SW disturbances. In addition to that, small scale SW structures measured at L1 can differ significantly from the structures that actually arrive at the magnetosheath (Borovsky, 2020). This would again change the upstream dynamic pressure threshold to a value that should not be compared to the dynamic pressure measured in the Earth's magnetosheath. Therefore, we compiled the second jet list using local magnetosheath dynamic pressure. We see that the median time lengths of jets detected by the local criteria are more uniform during different types of SW structures (Figure 3). We find that the number of extreme outliers in the jet data is considerably lower for the local jet list compared to the upstream jet list. While the upstream jet list is certainly valid for quiet and undisturbed SW times, we conclude that the local jet criteria are more reliable when analyzing times of SW disturbances. We find that the general trends in our results are the same for both jet definitions even with the previously mentioned shortcomings.

## 5 Summary and Conclusion

In this work we studied the connection between large‐scale SW structures and magnetosheath jets. To achieve this goal, we analyze the overlapping times of magnetosheath observation from THEMIS with times of SW events. We compile two jet lists by applying upstream and local threshold definitions using THEMIS magnetosheath observations. Sudden changes in SW parameters can suddenly change the detection threshold. Therefore, two jet definitions help us mitigate errors arising from a bias in the jet detection. We use a CME list compiled by Richardson and Cane (2010) for the start and end times of CME‐sheath and CME‐magnetic ejecta. For SIRs and HSSs we compile, unify, and expand times from several sources (Geyer et al., 2021; Grandin et al., 2019; Jian et al., 2011). The final SIR and HSS list includes SIR start times, HSS peak times, and HSS end times from 1995 to 2020.

First we check, how many detected jets are overlapping with large‐scale SW structures. Based on this analysis, we look at each spacecraft individually. In the second step, we calculate how the total time of observed magnetosheath jets time change during SW events. We look at SIR + HSS, CME‐sheath, and CME‐ME individually. In the last step, we use SEA analysis to determine, how the jet occurrence changes during SW events in general.

We find a relative difference in jet percentage during different types of large‐scale SW events. This is primarily a result of differences in jet numbers rather than due to a difference in mean jet duration. The number of observed jets within the Earth's magnetosheath increases during the passage of SIR and HSS by up to 50%. The number of jets decreases during the passing of a CME‐ME and its associated sheath by roughly 50%. Both our jet lists focus on dynamic pressure enhancements in the GSE‐X direction only. Therefore, these jets are more likely to reach the magnetopause, where they can potentially be geoeffective. This suggests that the number of geoeffective jets can be increased during SIR and HSS. For CMEs, while usually being significantly geoeffective themselves, the number of associated geoeffective jets seems to be low. Further statistical analysis to check differences in SW parameters for jets during each type of event is necessary. In addition to that, case studies will help us to gain in‐depth knowledge on individual effects happening in the magnetosheath during the passage of these types of events.

## Data Availability

We thank C. W. Carlson and J. P. McFadden for use of ESA data. We acknowledge the use of NASA/GSFC's Space Physics Data Facility's OMNI data and web services (https://omniweb.gsfc.nasa.gov/html/omni_min_data.html). THEMIS and OMNI data were accessed using the SPEDAS software (Angelopoulos et al., 2019). Both original jet lists covering the time ranges of 2008–2012 and 2012–2018 are available online https://osf.io/gf732/ (Plaschke, Hietala, & Angelopoulos, 2020), https://osf.io/7rjs4/ (Plaschke, Hietala, & LaMoury, 2020). We provide the new jet lists (upstream and local criteria) as well as the magnetosheath times at https://osf.io/6ywjz/ (Koller et al., 2021).
